# Temporal trends in adolescent sport participation in the south-east of France from 2001 to 2019

**DOI:** 10.1093/eurpub/ckac130.192

**Published:** 2022-10-25

**Authors:** M Luiggi, M Travert, C Gatouillat, J Griffet

**Affiliations:** 1 ADEF, Aix-Marseille University, Marseille, France; 2 ISM, CNRS, Aix-Marseille University, Marseille, France

## Abstract

**Background:**

Adolescents playing sport are more likely to reach the recommended levels of physical activity. In 2001, 2006, 2011 and 2019, four successive French national plans were launched to “develop physical and sports activity and limit sedentary living”. Monitoring sport participation rates (SPR) is one of the essential components to evaluate these plans. To date, information on temporal trends in SPR has mainly come from the national population. However, due to sample size, it was impossible to measure trends among adolescents on the territory level. Given the various economic and geographical disparities between territories, it is likely that territory specific trends exist. The main objective of this study was to measure temporal trends in adolescent SPR in the third biggest French department (South-East of France).

**Methods:**

Four retrospective studies were conducted in high-schools between March and April 2001, 2008, 2015 and 2019 (n = 4367). A quota sampling design was used to obtain geographically and socially representative samples. They were invited to report their sex, their socioeconomic status (SES) and their sport participation. A sports player was defined as an adolescent playing sport for at least one hour a week. SPR were calculated by sex and SES with 95% confidence interval (95% CI).

**Results:**

A decline in SPR, from 79.0% (95% CI = 76.4-81.7) to 64.5% (95% CI = 61.7-67.3), accompanied with a growth of social inequalities, were observed. SPR of low-SES adolescents declined from 67.7% (95% CI = 61.1-74.3) to 42.6% (95% CI = 36.7-48.4). SPR also declined from 72.5% (95% CI = 66.2-78.8) to 69.9% (95% CI = 64.5-75.3) for high-SES girls, from 87.2% (95% CI = 82.5-92.0) to 65.2% (95% CI = 59.6-70.9) for low-SES boys and from 91.0% (95% CI = 87.0-95.0) to 83.0% (95% CI = 78.5-87.4) for high-SES boys.

**Conclusions:**

Temporal trends in adolescent SPR in this territory are in decline since 2001. Governmental plans to improve SPR seems to have had a limited success in this territory.

**Key messages:**

- Weekly sport participation is declining among adolescents in the South-East of France.
- Social inequality in weekly sport participation are increasing over time.

